# The sponge holobiont in a changing ocean: from microbes to ecosystems

**DOI:** 10.1186/s40168-018-0428-1

**Published:** 2018-03-09

**Authors:** L. Pita, L. Rix, B. M. Slaby, A. Franke, U. Hentschel

**Affiliations:** 1 RD3 Marine Microbiology, GEOMAR Helmholtz Centre for Ocean Research, Kiel, Germany; 2 Christian-Albrechts-University of Kiel (CAU), Kiel, Germany

**Keywords:** Sponges, Holobiont, Health, Symbiosis, Microbiome, Nested ecosystems, Stress, Climate change, Dysbiosis, Disease

## Abstract

The recognition that all macroorganisms live in symbiotic association with microbial communities has opened up a new field in biology. Animals, plants, and algae are now considered holobionts, complex ecosystems consisting of the host, the microbiota, and the interactions among them. Accordingly, ecological concepts can be applied to understand the host-derived and microbial processes that govern the dynamics of the interactive networks within the holobiont. In marine systems, holobionts are further integrated into larger and more complex communities and ecosystems, a concept referred to as “nested ecosystems.” In this review, we discuss the concept of holobionts as dynamic ecosystems that interact at multiple scales and respond to environmental change. We focus on the symbiosis of sponges with their microbial communities—a symbiosis that has resulted in one of the most diverse and complex holobionts in the marine environment. In recent years, the field of sponge microbiology has remarkably advanced in terms of curated databases, standardized protocols, and information on the functions of the microbiota. Like a Russian doll, these microbial processes are translated into sponge holobiont functions that impact the surrounding ecosystem. For example, the sponge-associated microbial metabolisms, fueled by the high filtering capacity of the sponge host, substantially affect the biogeochemical cycling of key nutrients like carbon, nitrogen, and phosphorous. Since sponge holobionts are increasingly threatened by anthropogenic stressors that jeopardize the stability of the holobiont ecosystem, we discuss the link between environmental perturbations, dysbiosis, and sponge diseases. Experimental studies suggest that the microbial community composition is tightly linked to holobiont health, but whether dysbiosis is a cause or a consequence of holobiont collapse remains unresolved. Moreover, the potential role of the microbiome in mediating the capacity for holobionts to acclimate and adapt to environmental change is unknown. Future studies should aim to identify the mechanisms underlying holobiont dynamics at multiple scales, from the microbiome to the ecosystem, and develop management strategies to preserve the key functions provided by the sponge holobiont in our present and future oceans.

## Background

Marine animals live and evolve in a sea of microbes. The ocean is the largest habitat on our planet and microbes are its most abundant inhabitants. These microorganisms (i.e., viruses, bacteria, archaea, microeukaryotes) play a key role in global biogeochemical cycles [1]; yet, scientists are only beginning to reveal their genomic and metabolic diversity [2]. Marine microbes exist not only in a planktonic state but also in symbiosis with macroorganisms: animals, plants, and algae alike [3, 4]. The prevalence of these associations implies that multicellular organisms can no longer be considered as autonomous entities [5] but rather as holobionts (syn. “metaorganisms” [6]), encompassing the host plus its associated microbiota [7, 8]. The microbial partners contribute to the nutrition [9], defense [10], immunity [11], and development [12] of the host; thereby collectively influencing its health and functioning.

The first approaches to define the holobiont consisted of characterizing the set of microbial taxa common to all individuals of a certain species, the core microbiota. Later definitions, enabled by massively increased sequencing efforts, included the core set of functional genes that ensured homeostasis of the holobiont [13, 14]. However, holobiont functioning is not only determined by the processes carried out by the individual members, but also by the interactions among them. Consequently, holobionts can be regarded as complex ecosystems to which the concepts and methodologies from ecology can be applied to understand the drivers of holobiont stability [15–17]. Under this perspective, the holobiont represents a dynamic equilibrium characterized by two important properties: resistance (the ability to withstand perturbation unchanged) and resilience (the capacity to recover upon disturbance). This view contributes to understanding the dynamics of the microbial and host-related processes involved in maintaining a healthy holobiont [15–17] and its response to environmental change [18–20].

Moreover, the holobiont performs functions that cannot be accomplished by the partners separately. The microbiome provides essential functions to the host and together they mediate the interactions of the holobiont with the surrounding organismal community [5]. Through cascading effects, the microbiome can ultimately impact ecosystem health and functioning. One prominent example is the symbiosis between corals and their photosynthetic dinoflagellates (*Symbiodinium* spp.). By virtue of autotrophic CO_2_ fixation, *Symbiodinium* provide the necessary primary metabolism that enables corals to engineer the three-dimensional calcium carbonate structure that, ultimately, supports the entire reef ecosystem [21]. Conversely, coral bleaching resulting from the loss of dinoflagellate symbionts not only has severe consequences for coral health, but also has devastating effects on the entire coral reef ecosystem [22]. Therefore, an integrative approach that considers these different scales is needed to evaluate holobiont function within these nested ecosystems.

In this review, we will discuss how host- and microbial-mediated activities translate into the emerging functions of the sponge holobiont that impact the surrounding ecosystem, and how the holobiont is in turn affected by the anthropogenic pressures increasingly impacting marine ecosystems. Marine sponges (phylum Porifera) perfectly illustrate the idea of holobionts as ecosystems, given the exceptionally diverse microbial communities housed within them [23, 24]. Sponges are a successful (> 8000 species) and evolutionarily ancient phylum, their members being globally distributed and abundant within the benthic communities of a wide range of habitats [25, 26]. Their sessile filter-feeding lifestyle constantly exposes them to the microbes in the seawater that form their primary food source; yet, they harbor distinct symbiotic microbial communities. Sponges influence ecosystem functioning by modifying biotic and abiotic factors (reviewed in [27]). For example, they provide habitat for a wide range of fauna and play an important role in benthic-pelagic coupling due to their impressive filtering capacity [26, 28–30]. The field of sponge microbiology has consolidated in recent years as collaborative efforts have developed standardized protocols and curated databases on sponge-associated microbial diversity (i.e., the Global Sponge Microbiome Project) [23, 24]. Novel approaches, combined with state-of-the-art techniques, have begun to reveal the functions of the collective microbial community and individual symbiont groups [31–35]. One major finding is that many of the functional roles provided by sponges are indeed mediated by their associated microbes. The natural variability across sponge holobionts and environments, together with the possibility for lab experiments, opens up the opportunity to address the dynamics of these complex symbiotic systems [36]. It is therefore timely to scale up the marine sponge holobiont concept from the microbial to the ecosystem level, particularly in the context of health, disease, and response to anthropogenic pressures.

## The marine sponge holobiont

### Microbial core diversity

**Fig. 1:**
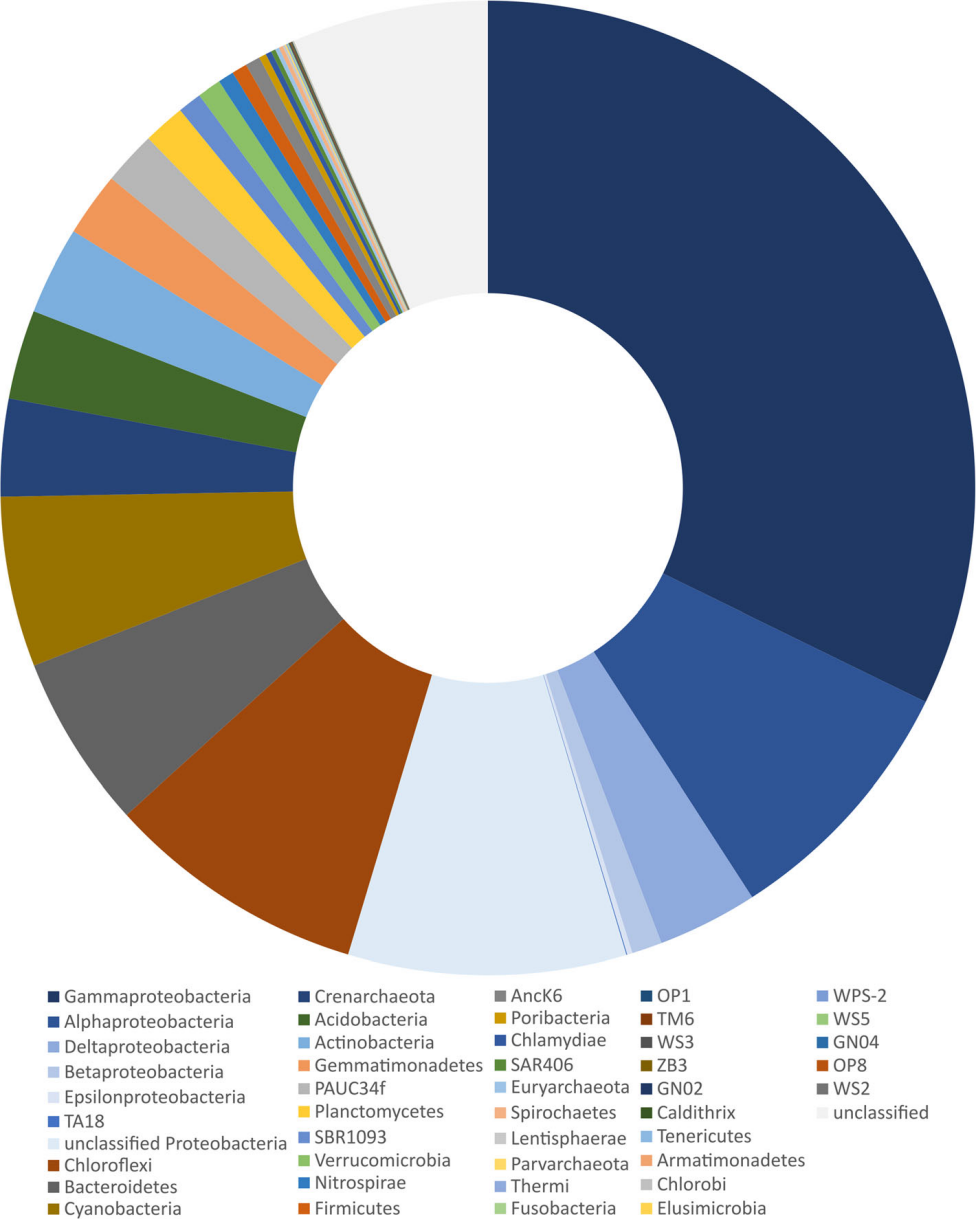
Microbial OTU richness in sponge-associated microbial communities at phylum level. The Greengenes annotation of the representative sequences for sponge-associated OTUs detected by the Global Sponge Microbiome [23] was used to create this chart. A diversity of 43,034 OTUs from 39 classified microbial phyla (bacteria and archaea) was detected in the microbiomes of the 81 sponge species in this project [23]

The Global Sponge Microbiome Project, under the umbrella of the Earth Microbiome Project, is a recent collaborative initiative to assess the microbial diversity in sponges from around the world, following standardized protocols [23, 24]. Similar to the Human Microbiome Project [37], the main goal was to create a publicly available database that would enable comparative studies in order to discover common patterns and principles of sponge-associated microbial assemblies. The first comprehensive study [23], including 81 sponge species, revealed that the sponge microbiome spans at least 39 microbial phyla and candidate phyla (Fig. 1). The most dominant bacterial symbiont groups belong to the phyla *Proteobacteria* (mainly *Gamma-* and *Alphaproteobacteria*), *Actinobacteria*, *Chloroflexi*, *Nitrospirae*, *Cyanobacteria*, and candidatus phylum *Poribacteria*, while *Thaumarchaea* represents the dominant archaeal group [23, 24]. The microbial communities are species-specific, but composed of both generalist microbes that are detected in the majority of sponge species from diverse geographic regions, as well as specialists that are enriched in particular species but are rare or absent in most other species [23, 38]. A second sequencing effort has recently expanded this dataset to over 260 sponge species, yet, the overall patterns remain consistent [24].

In terms of community structure, complex host-associated microbial communities are divided into a core microbiome (members that are highly prevalent in all host individuals of the same species) and a variable microbiome (members of the microbial community that are recovered only from some individuals or that vary in their relative abundance) [39]. Surveys along different environmental gradients (e.g., geographical distance [40], season [41, 42], depth [43], and habitat [44]) have consistently confirmed that sponges harbor species-specific and stable microbiomes at different prokaryotic taxonomic levels [45] and prevalence thresholds [46]. This stability is remarkable when compared with the dynamic turnover of bacterioplankton in the surrounding water, upon which sponges feed [41], and hints to the importance of host-related factors in shaping the core microbiome. However, there is also evidence that suggests that environmental conditions impact sponge-associated microbial diversity, particularly the variable fraction. For example, two sponge species that were able to colonize and proliferate in the acidified environment of a CO_2_ seep [47], harbored significantly higher relative numbers of symbiotic *Synechococcus* at the CO_2_ seep compared with specimens at control sites less than 500 m away. Temporal variation, depth, and habitat type can also impact the composition of sponge-associated microbiota [43, 48, 49]. An additional driver of sponge-associated microbiota structure is the HMA-LMA dichotomy.

Microbe-microbe interactions within the holobiont can further affect the dynamics and stability of the symbiosis [50, 51]. Network and modeling analyses aim to disentangle the strength and nature (positive, negative, or neutral) of the interactions and predict their dynamics. Bacteria-bacteria network analysis of the core microbiota in different sponge species has revealed a low connective network with very few strong and many weak unidirectional interactions (i.e., amensalism (−/0) and commensalism (+/0) prevailed over cooperation (+/+) and competition (−/−)) [23]. These findings are consistent with mathematical models that predict that weak and non-cooperative interactions help to stabilize highly diverse microbial communities, whereas cooperation yields instability in the long term by fueling positive feedbacks [52].

### Microbial core functions

Since sponge symbionts remain largely uncultivable, culture-independent methodologies have been instrumental to gain genomic and thereby putative functional information on sponge symbionts. Indeed, a variety of metagenomic, metaproteomic, and metatranscriptomic sequencing approaches have been employed to elucidate the functions of the sponge microbiome [31, 53–56]. Single-cell genomics and metagenomic binning have obtained a number of individual symbiont genomes [33, 57–59]. Furthermore, novel visualization techniques have been developed and applied to test hypotheses derived from genomic data as well as gain valuable spatial information [34, 35].

Comparisons between metagenomes of sponge-associated and seawater microbial consortia have identified gene features enriched in sponge symbionts that might be relevant to the symbiosis [31, 53, 60, 61]. These features have been found in the microbiomes of multiple sponge species from various geographic regions, but they are mediated by different microbial taxa and carried out by different, although analogous, pathways [53, 62]. This functional convergence hints to features that are necessary for microbial persistence in the host as well as holobiont success, and therefore can be considered core functions of the sponge microbiome [23, 31, 53, 62]. Beyond the housekeeping genes required for microbial life, we define core functions as the range of metabolic and defensive features that allow the sponge microbiota to colonize, interact with, and adapt to the host environment (Table 1). Metabolic features within the core functions include (a) the autotrophic and heterotrophic pathways for symbionts to utilize the nutrients available in the sponge host environment—either produced by the host itself or filtered in from the surrounding seawater—and (b) the pathways that directly contribute to the symbiotic relationship with the host. Defensive features include those that enable symbiont persistence within the sponge host. In addition, most studied sponge symbiont genomes lack genes encoding for flagella, which points to a non-motile existence within the mesohyl matrix [33, 61] (but see [53, 63]).

**Table 1:** Core functions of the sponge microbiome

	Core function	Interpretation	Reference
Metabolic features	Nitrogen metabolism with emphasis on ammonia oxidation	Utilization of environmental and host-derived nutrients	Reviewed in [77]
	Carbon metabolism with emphasis on complex carbohydrates	Utilization of environmental and host-derived nutrients	[32, 33]
	Nitrogen and carbon metabolism utilizing creatinine	Utilization of environmental and host-derived nutrients	[35, 53]
	Vitamin synthesis (especially thiamine and vitamin B12)	Overproduction of vitamins that are then utilized by the sponge host	[31, 53, 72]
	Secondary metabolism	Microbe-microbe interaction, defense of the holobiont	
	Carnitine (vitamin BT) utilization	Utilization of host-derived component	[33]
Defense features	CRISPR-Cas systems	Defense against viruses/phages	[31, 33, 61]
	Toxin-antitoxin systems	Defense against foreign DNA	[31, 33, 61]
	Restriction modification systems	Defense against foreign DNA	[31, 33, 61]
	Eukaryotic-like protein domains	phagocytosis evasion	[31, 54, 81–83]
	Modifications of the lipopolysaccharide	phagocytosis evasion	[83, 84]
Other	Mobile genetic elements and transposases	Increased levels of horizontal gene transfer	[31, 53, 61, 85]

Nitrogen is generally a limiting nutrient in the marine environment but is excreted in large quantities by the sponge host, which produces ammonia as a metabolic waste product. Consequently, it is not surprising that sponge symbionts are enriched in nitrogen metabolism genes [53, 64–66]. Ammonia oxidation is particularly prevalent and predominant [53, 62, 64, 67, 68], but most major nitrogen cycling pathways occur, including both aerobic (e.g. nitrification, nitrogen fixation) and anaerobic (e.g., denitrification, anammox) processes [59, 64, 69–73]. The presence of anaerobic metabolism is likely facilitated by the fact that the sponge tissue can rapidly become anoxic during temporary cessation of sponge pumping [74–76].

A large part of the sponge microbiota relies on heterotrophic metabolism and uses nutrient sources derived from the seawater filtered by the sponge, as well as produced by the sponge host itself [77]. With respect to carbon metabolism, the degradation of complex carbohydrates appears to be a dominant feature in sponge symbioses and highlights the role of heterotrophy in these communities [32, 33]. For example, there is mounting evidence that the symbionts also feed on sponge cell biomass and components of the sponge extracellular matrix [32, 33, 78]. The core functions of the microbiota also encompass metabolic features that potentially benefit the host. Sponge symbionts are enriched in genes related to the synthesis of vitamins, such as vitamin B_1_ and vitamin B_12_ [31, 53, 72, 79]_,_ suggesting the symbionts may satisfy the host’s demand for these essential vitamins. For example, a recent holobiont transcriptome study showed that the sponge microbiome was enriched in gene functions related to anabolic pathways of several amino acids and vitamins for which the host *Xestospongia muta* expressed only catabolic reactions [72]. The diverse and abundant range of membrane transporters (e.g., ABC-type transporters) encoded by the sponge microbiome provides mechanisms to facilitate these putative metabolic exchanges [53]. In addition, microbial symbionts have been identified as the source of certain secondary metabolites that constitute the chemical defense of the sponge holobiont [79, 80].

In order to persist within sponges, microbes must avoid phagocytosis by the host cells. Eukaryotic-like protein domains (ELPs), such as ankyrin repeat proteins, tetratricopeptide repeat proteins, and leucine-rich repeat proteins, were found to be highly enriched in and also expressed by sponge symbionts [31, 54, 81–83]. ELPs mediate protein-protein interactions and are hypothesized to play a role in the evasion of phagocytosis [81]. Another possible strategy has been found in the cyanobacterial symbiont “*Candidatus* Synechococcus spongiarum” that lacks a lipopolysaccharide (LPS) antigen [83]. This modification of the LPS in the symbiont vs free-living *Synechococcus* could represent a mechanism for the sponge host to discriminate between food and symbiotic bacteria [84].

Additional defensive core functions relate to protection and stress response (e.g., stress proteins, restriction modification, toxin-antitoxin systems, and clustered regularly interspaced short palindromic repeats CRISPRs). These defensive functions likely shield sponge symbionts against incoming foreign DNA, pathogens, and toxins to which they are exposed due to the pumping activity of the host [31, 33, 61]. Interestingly, elevated GC content and larger genome sizes were observed in sponge metagenomes in comparison to seawater metagenomes [61]. The larger genome sizes are attributed to higher levels of horizontal gene transfer (HGT) within the sponge host than in the seawater environment and adaptations to the more variable and nutrient-rich sponge-associated environment [61]. The hypothesis of increased levels of HGT is supported by the high number of mobile genetic elements found in the genomic repertoires of sponge symbionts, as well as transposases necessary for genetic transfer, which likely played a role in the evolutionary adaptation of the sponge microbiota to the symbiotic lifestyle [31, 53, 85].

To elucidate their role within the community, single members of the sponge microbiome have been studied individually and revealed examples of specialization. For example, metagenomic binning revealed three symbiont guilds in *Aplysina aerophoba* displaying metabolic specialization to different substrates [33]. Each guild was composed of a phylogenetically diverse group of symbiont members, suggesting independent evolution to different micro-niches within the sponge extracellular matrix. A remarkable example of a function carried out by a specific member of the microbiota is that of “*Candidatus* Entotheonella factor,” which produces almost all polyketides and peptide families that were previously attributed to synthesis by the host sponge, *Theonella swinhoei* [79]. This example is exceptional in that a specific bacterial clade associates with a specific sponge host and endows the holobiont with defensive capacities. Moreover, a recent study merging metagenomic binning, metatranscriptomics and visualization techniques has revealed tightly interlinked metabolic pathways between members of the holobiont of the *Cymbastella concentrica*; two proteobacteria, a thaumarchaeon and a diatom [35]. The thaumarchaeon and a *Nitrospira* bacterium are hypothesized to be coupled in their nitrification activity, producing nitrate that is subsequently used by the *Phyllobacteriaceae* bacterium and the diatom [35].

Studies on single symbiont groups highlight the potential for high levels of specialization and interdependency within sponge holobionts. They also complement community level approaches by linking diversity with function. In these complex ecosystems, it is particularly challenging to identify which taxa contribute to each functional trait and the degrees of redundancy of particular functions remains unknown. Future studies should validate the genomic information presented here with a focus on those functions directly involved in the symbiotic interaction. In this direction, further efforts for cultivation would provide valuable insight into the chemical characterization and environment-regulated activity of target symbionts.

### The sponge host

Sponge hosts may be viewed as ecosystem engineers [52], since they provide a certain habitat that selects for the presence and persistence of certain microbes, but not others. They also control their microbial residents by specifically recognizing and differentiating between foreign and symbiotic microbes [84, 86], likely via the innate immune system. The innate immune system, traditionally investigated in the context of pathogenesis, allows colonization and long-term maintenance of the symbiosis (reviewed in [87]). Pattern recognition receptors (PRRs) sense microbial ligands, but the activated response is context-dependent: symbiont-derived signals promote homeostasis, whereas pathogens induce an inflammatory response.

The underlying molecular mechanisms of microbial recognition by sponges remain elusive due to experimental limitations [36]. However, high-throughput sequencing data revealed that sponges harbor a complex genomic repertoire encoding a broad spectrum of immune receptors (including Toll- and NOD-like receptors and scavenger receptor cysteine-rich (SRCR) family members) [60, 88, 89], for which the role in responding to microbes is beginning to be elucidated [90, 91]. For example, the sponge *Petrosia ficiformis* displayed an increased expression of a gene containing the conserved SRCR domain when living in symbiosis with a cyanobacterium, in comparison to the aposymbiotic status [90]. Also, components of the Toll-like receptor pathway such as MyD88 were involved in the response to microbial signals in different species [91, 92]. In a recent experiment on juvenile *Amphimedon queenslandica* [91], bacterial encounter involved regulation of SRCR-containing genes, but the downstream response differed depending on the origin of the bacteria. In particular, the transcription factors FoxO and NFκβ were upregulated upon exposure to own symbionts, but not to a bacterial fraction from another sponge species. These new findings suggest that sponges actively recognize and discriminate microbes via immune signaling. Host recognition of the microbiota also acts on symbiont acquisition: the host promotes certain microbial species through vertical transmission from adult to progeny or by direct recognition and uptake of symbionts from the environmental pool. In sponges, both modes of microbial transmission likely occur [93–98], yet the underlying mechanisms of host-microbe crosstalk remain to be identified. Host-related processes would impose a means to maintain specific microbiomes, but it is likely that host-independent process (e.g., stochasticity) also play a role, particularly in the environmentally acquired microbial fraction [18].

## From microbes to ecosystems

**Fig. 2:**
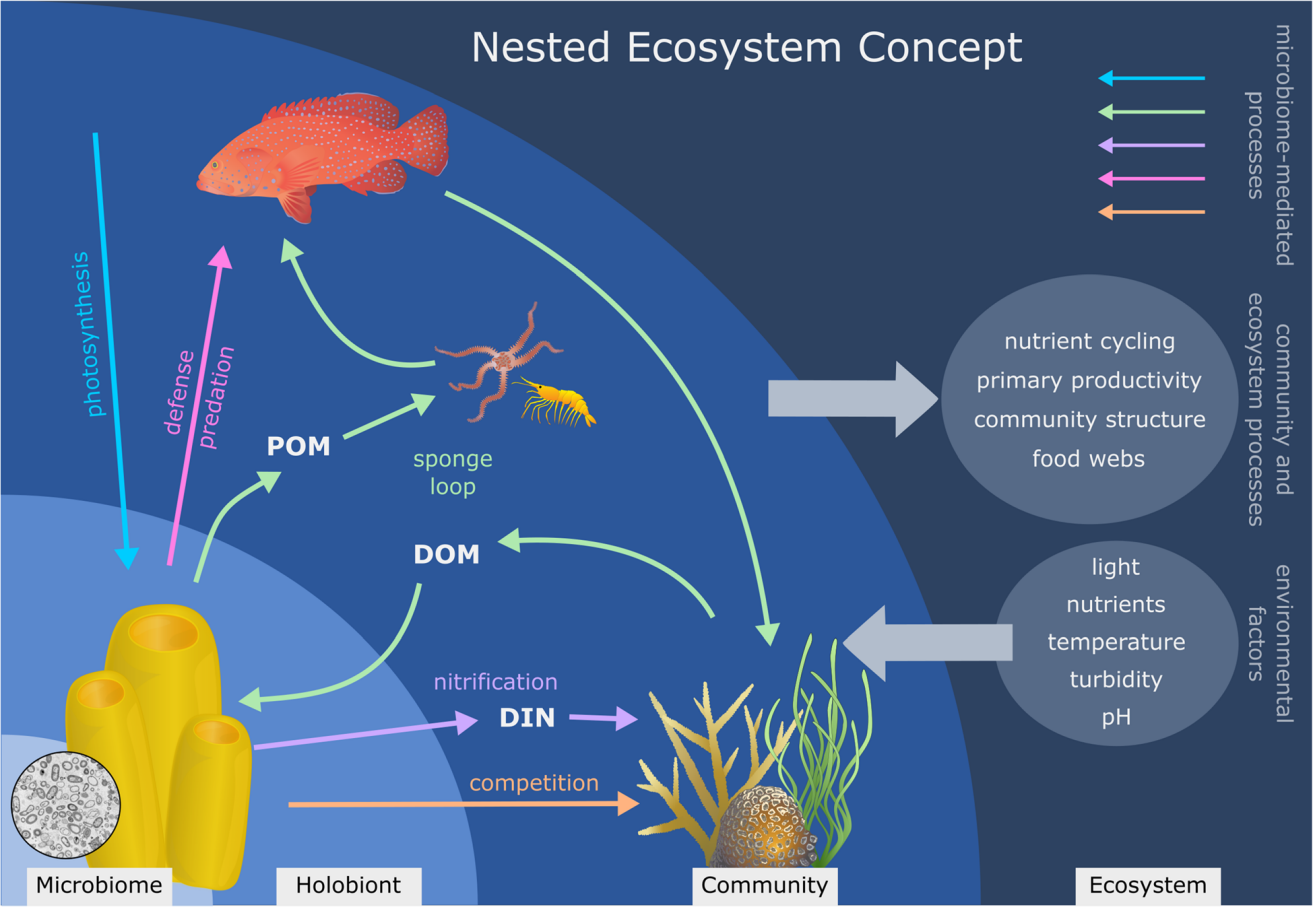
The sponge holobiont as an example of the concept of nested ecosystems. Key functions carried out by the microbiome (colored arrows) influence holobiont functioning and, through cascading effects, subsequently influence community structure and ecosystem functioning. Environmental factors act at multiple scales to alter microbiome, holobiont, community, and ecosystem scale processes. Thus, factors that alter microbiome functioning can lead to changes at the holobiont, community, or even ecosystem level and vice versa, illustrating the necessity of considering multiple scales when evaluating functioning in nested ecosystems. DOM, dissolved organic matter; POM, particulate organic matter; DIN, dissolved inorganic nitrogen

Highly diverse holobionts can be considered as complex ecosystems [15, 20, 52] in which the actions and interactions of the various members shape the overall functioning of the holobiont. These individual ecosystems in turn interact with and influence neighboring holobionts, such that they are further integrated into larger communities and ecosystems that interact at successively larger scales [5]. Consequently, the actions of a single member of the microbiota can exert an effect far beyond that of the holobiont. Key examples of this concept of “nested ecosystems” are the chemoautotrophic symbionts associated with hydrothermal vent animals or the phototrophic symbionts associated with reef-building corals that supply nutrition for their hosts who in turn support the larger communities in these unique ecosystems [5, 22, 99]. The sponge microbiome provides a number of functions that are amplified by host activity and through cascading effects mediate the functioning of the sponge holobiont at the community and ecosystem level. Here, we provide five key examples where such microbial-mediated functions scale up to influence community structure and contribute to ecosystem primary productivity, biogeochemical nutrient cycling, and benthic food webs (Fig. 2).

### Photosynthesis

Sponges can host photoautotrophic symbionts that not only contribute to host nutrition through the translocation of photosynthetically fixed carbon and nitrogen [100–102], but also contribute to ecosystem primary productivity [103]. In species hosting photosymbionts, these symbionts can potentially supply more than 50% of the holobiont’s energy requirements [102–104]. Symbiont contribution to host nutrition appears to be highly variable as only some sponges receive a nutritional benefit from their photosymbionts, and reduced photosynthetic capacity does not always correspond with a reduction in host growth [102, 105–107]. Nevertheless, photosynthetic symbionts enable sponge holobionts to contribute to the gross primary productivity that supports the entire ecosystem [108]. Similarly, evidence for chemoautotrophy [109–111] suggests that chemoautotrophic sponge holobionts may contribute to ecosystem primary productivity, particularly in deep-sea environments, such as hydrothermal vents and cold seeps [68, 111–113], where such symbioses are essential for supporting life in these extreme environments [99].

### The sponge loop

The assimilation of dissolved organic matter (DOM) by sponge holobionts facilitates DOM cycling in benthic habitats with cascading effects on marine food webs [29]. Microbes contribute to the assimilation of DOM by the sponge holobiont [29, 114, 115], which can account for up to ~ 90% of the holobiont’s total heterotrophic carbon uptake [116–122]. In addition to providing an important food source for the holobiont, DOM uptake by sponges has been proposed to play a key role in DOM cycling within tropical and deep-sea coral reefs via a pathway termed the “sponge loop” [29, 114]. By rapidly taking up the DOM released by primary producers and converting it into particulate organic matter (POM) in the form of detritus, sponges transform DOM into a food source that is more readily available to other benthic reef fauna [29, 115, 123, 124] (Fig. 2). Similar to the microbial loop [1, 125], the sponge loop therefore enables the energy and nutrients in DOM to be retained and recycled within reef food webs. Although exact quantification of DOM cycling by the sponge loop is lacking, DOM uptake by cryptic sponges in the Caribbean and Indo-Pacific is estimated to be on the same order of magnitude as gross reef primary productivity and may even exceed DOM cycling by the microbial loop [29, 126]. Thus, by acting through the sponge loop, the sponge microbiome may play an important role in driving DOM cycling at the ecosystem level, as well as facilitating energy transfer through reef food webs (Fig. 2).

### Inorganic nutrient cycling: nitrogen and phosphorous

Sponge holobionts play an important role in the biogeochemical cycling of nitrogen—one of the main nutrients limiting primary productivity in the marine environment [30, 127]. This capacity for nitrogen cycling is intimately linked to nitrogen transformations carried out by the sponge microbiome [72, 128]. Nitrification via ammonia and nitrite oxidization is particularly prevalent and may benefit the host through removal of the large quantities of host-excreted ammonia [129]. Whether a sponge hosts large numbers of highly active nitrifying microbes dictates if it releases nitrogen primarily as ammonia or nitrate [62, 119, 127]. Moreover, since the sponge microbiome can simultaneously perform competing nitrogen cycling pathways (e.g., nitrification and denitrification) [69, 72, 75], the relative activities of different members of the microbiome can further influence whether the holobiont acts as a net source or sink of bioavailable nitrogen [101, 128]. In oligotrophic marine environments like coral reefs, nitrogen can be released by sponges at ecologically relevant quantities [127, 130] and can facilitate the growth of nearby primary producers such as corals and algae [131, 132]. Sponge-associated microbes are also involved in the cycling of other key limiting nutrients such as phosphorous. While sponges have been shown to release inorganic-phosphorous in the form of phosphate [62, 119, 133, 134], the discovery of abundant intracellular polyphosphate granules in the microbial symbionts of three phylogenetically distinct reef sponge species suggests the sponge microbiome may also mediate a pathway for phosphorous sequestration on coral reefs [135]. These microbial-generated storage granules can account for up to 40% of the total sponge phosporous content, and thus may substantially influence phosphorous availability in habitats with high sponge biomasses [135, 136]. The sponge microbiome therefore influences both the quantity and speciation of inorganic nutrients made available to neighboring primary producers in coral reefs and other benthic ecosystems (Fig. 2).

### Chemical defense and predation

The sponge microbiome also conveys defensive capacities to the host that strongly influence the interactions between sponges and other organisms within benthic communities (Fig. 2). Sponge holobionts produce a diverse array of secondary metabolites with antiviral, antimicrobial, cytotoxic, allelopathic, and antipredatory effects [10, 129, 137, 138], some of which have been attributed to the microbiome [79, 80, 139]. The production of biologically active feeding deterrent compounds is a common defensive strategy employed by sponges to avoid predation [140–142]. One of the earliest studies to link a sponge-derived secondary metabolite to the microbiome found that compounds isolated from the cyanobacterial symbionts of the sponge *Lamellodysidea herbacea* deterred fish feeding [143]. Subsequent studies have found increasing evidence that the microbiome is actively involved in the production of bioactive compounds with putative anti-predatory effects in a range of chemically defended sponge species [10, 79, 144, 145]. Predation is a major process governing benthic community structure and can alter sponge community composition at sub-meter to habitat scales [146–148]. In habitats with high abundances of sponge predators, sponges without chemical defenses may be entirely excluded [148–150]. By influencing holobiont susceptibility to predation, the sponge microbiome thereby influences benthic community structure.

### Competition

Spatial competition is another important biotic factor structuring benthic communities, and the sponge microbiome can mediate such interspecific interactions through a combination of metabolic and chemical defensive functions that enhance the competitive capacity of the holobiont (Fig. 2). For example, the abundant cyanobacterial symbionts of the coral-killing sponge *Terpios hoshinota* [151] play a key role in enabling the *Terpios* holobiont to aggressively overgrow a wide range of coral species [152, 153]. They not only provide cytotoxic secondary metabolites [154] but also photosynthates that enhance the physiological performance of the host [153, 155]. Impairing the photosynthetic capacity of the symbionts through shading stops the growth of the sponge and prevents it overgrowing adjacent corals [153], demonstrating the importance of the symbionts in mediating these competitive interactions. Outbreaks of *Terpios hoshinota* have been implicated in causing widespread coral mortality [156, 157]. Consequently, this provides an example of how symbionts can dramatically influence holobiont competitiveness and thereby alter benthic community dynamics with catastrophic results.

These five examples highlight how the sponge microbiome can influence functioning at the holobiont, community, and ecosystem scale through the concept of nested ecosystems. Moreover, these microbiome-mediated functions are in turn shaped by environmental factors that also act on multiple scales [158] and feedback on the functioning of the holobiont by modifying sponge primary productivity, nutrient fluxes, and chemical defenses [134, 159–161]. Thus, future studies need to target the mechanisms behind host-symbiont interactions and link multiple scales if we are to unravel how the sponge microbiome may alter holobiont functioning under future environmental changes.

## Holobiont responses: stress, dysbiosis, and acclimatization

### Holobiont health

**Fig. 3:**
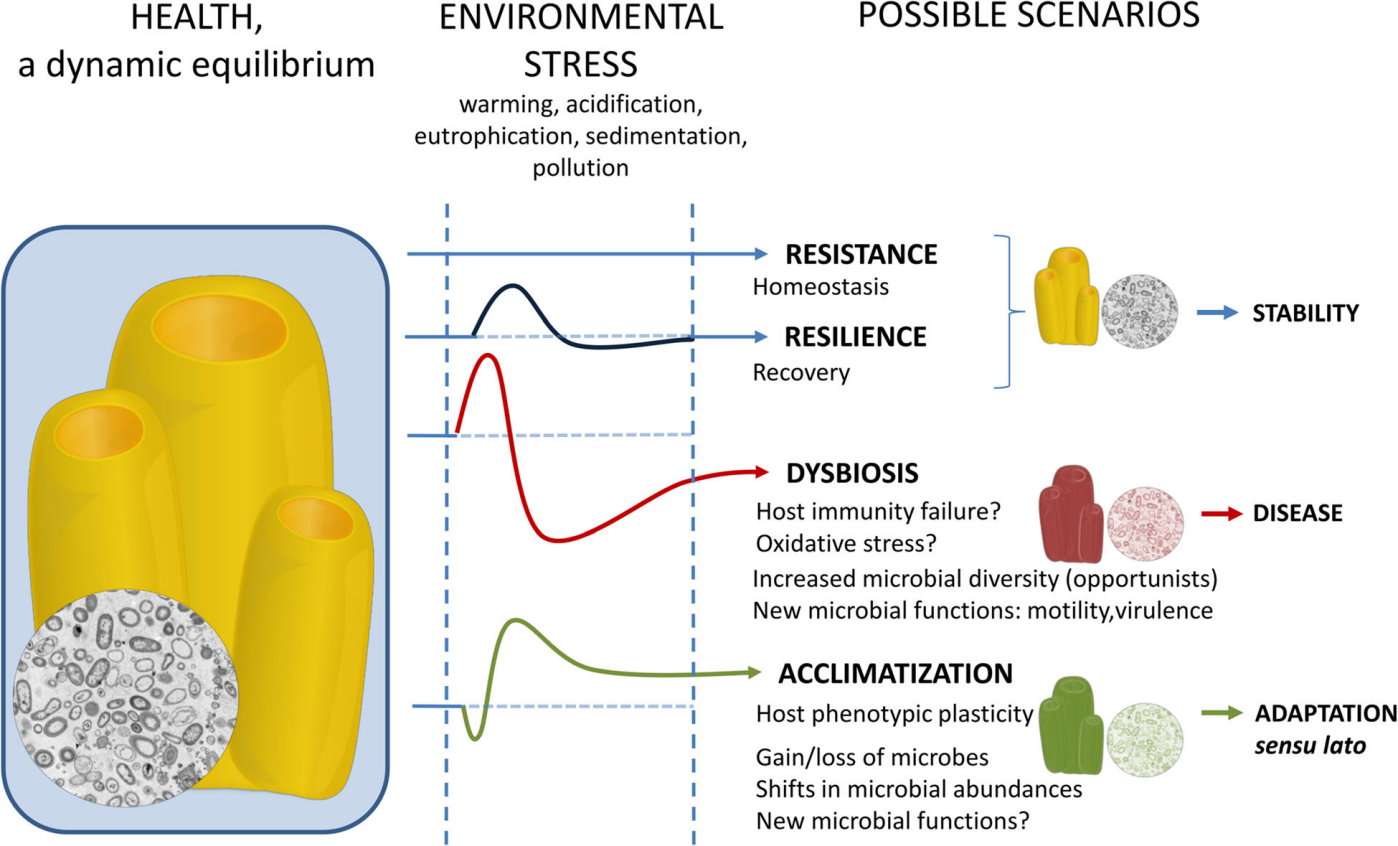
Conceptual representation of holobiont health and the potential outcomes upon environmental stress. Health is regarded as a dynamic equilibrium balanced by the host, the microbiome, as well as the interaction between them. Understanding the underlying principles of health and holobiont dynamics would help predict the responses upon perturbation and whether the final outcome will allow stability, yield disease, or turn into an opportunity for adaptation

The healthy holobiont is considered an ecosystem that is in a state of dynamic equilibrium. Like in any ecosystem, the strength and outcome (i.e., beneficial, neutral, or detrimental) of the interactions among the members of the holobiont may be affected by perturbations that challenge the healthy equilibrium (Fig. 3). Upon disturbance, alternative scenarios are possible. On the one hand, homeostasis can maintain healthy baseline conditions through mechanisms of resistance or resilience [162]. On the other hand, perturbations may disrupt the balance, leading to dysbiosis and, potentially, disease [163, 164]. Moreover, perturbations may act as a selective force (at the microbial, host, and/or holobiont level) so that the system reaches a new healthy state that allows it to better cope with environmental change (i.e., acclimatization). If the new features enhance holobiont fitness and can be transmitted to new generations, they may yield holobiont adaptation *sensu lato* [165]. The holobiont concept provides the framework to elucidate sponge responses to environmental change, the role of dysbiosis in disease, and the contribution of the microbiota to holobiont persistence.

### Stress and dysbiosis

Human activities are modifying the marine environment at a pace never before recorded [166]. Some of the major anthropogenic stressors threatening the oceans are climate change (ocean warming and acidification) and the deterioration of water quality (e.g., eutrophication, sedimentation, and pollution). But what are the consequences of these environmental stressors for sponge-associated microbial communities (Table 2)? Some studies report non-significant changes in the microbial community structure upon perturbations such as warming, increased sedimentation, or enriched nutrient concentrations, at least at sub-lethal stress levels [167–170]. The strength of the perturbations is therefore a major factor determining the ability of the holobiont to maintain a stable state. For example, the resilience of *Xestospongia muta* to bleaching (i.e., the ability to recover its cyanobacterial population) was only possible as long as stress was kept below a certain threshold [171]. Beyond these thresholds, significant shifts of the sponge-associated microbiota are commonly reported, mainly in already necrotic tissues as well as in apparently healthy tissues in contact with necrotic areas [158, 172].

**Table 2:** Response of sponge microbiome to environmental stressors

Sponge species	Microbial response			Host	Ref.
	Method	Diversity	Function	response	
OCEAN WARMING					
*Geodia barreti*	EMP pipeline	No	–	Ecophysiology	[168]
*Rhopaloeides odorabile*	TRFLP; metagenomics	Yes	Yes	RT-qPCR	[158]
*R. odorabile*	pyrosequencing on DNA and cDNA	Yes	–	–	[182]
*R. odorabile* larva	DGGE	Yes	–	–	[231]
*R. odorabile*	DGGE and cloning	Yes	–	–	[232]
*Ircinia fasciculata*, *I. oros*	TRFLP	No	–	–	[233]
*I. fasciculata*	PAM fluorometry	–	Yes	–	[194]
*Ianthella basta*	DGGE and cloning	Yes	–	–	[173]
*Xestospongia muta*	TRFLP, cloning and RT-qPCR of amoA gene	Yes	Yes	–	[234]
*Halichondria bowerbanki*	DGGE	Yes	–	–	[235]
OCEAN ACIDIFICATION					
*Dysidea avara*, *Agelas oroides*, *Chondrosia reniformis*	Pyrosequencing	spp-specific	–	Growth	[213]
*Coelocarteria singaporensis, Cinachyra sp*	Pyrosequencing; PICRUST	Cyano	yes	–	[47]
Ocean warming and ocean acidification					
*X. muta*	Pyrosequencing and PICRUST; PAM fluorometry	Yes	yes	–	[174]
*Carteriospongia foliascens*; *R. odorabile*; *Stylissa flabelliformis*; *Cymbastella coralliophila*	PAM fluorometry	–	yes	Ecophysiology	[183]
EUTROPHICATION, SEDIMENTATION, POLLUTION					
*C. foliascens*, *C. coralliophila; Cliona orientalis*, *Coscinoderma matthewsi*, *S. flabelliformis*	Illumina; PAM fluorometry	spp-specific	no	Ecophysiology	[170]
*Cymbastela stipitata*	Pyrosequencing on DNA and cDNA	No	–	–	[169]
*Haliclona cymaeoformis*	Pyrosequencing; metagenomics	Yes	yes	–	[176]
*I. basta*	DGGE	No	–	–	[173]
*R. odorabile*	RFLP; FISH	Yes	–	–	[236]
Ocean warming and eutrophication					
*R. odorabile*	DGGE and pyrosequencing; DGGE of amoA gene	No	–	–	[167]

Responses were assessed in aquarium experiments, except for reference [47]. Molecular analyses were performed on 16S rRNA gene, unless stated otherwise. *Fun.* function. *Ref* references, *Spp-specific* species-specific response. *EMP pipeline* standardized protocol applied during the global Sponge Microbiome Project following Earth Microbiome Project guidelines. *TRFLP* terminal restriction fragment length polymorphism, *RFLP* restriction fragment length polymorphism, *DGGE* denaturing gradient gel electrophoresis. *RT-qPCR* real-time quantitative PCR. *PICRUST* function was inferred from taxonomic diversity by PICRUST tool [237]. *PAM fluorometry* pulse amplitude modulated diving fluorometer as measurement of photosynthetic capacity

Stress can induce dysbiosis: a disruption of the symbiotic community diversity. In sponges, it is often characterized by an increased alpha diversity [173] and/or a shift from sponge-enriched microbes (closely related to other sponge symbionts) to opportunists (microbes closely related to free-living organisms) [167]. Even in the absence of significant changes in alpha-diversity, stress-related increases of beta-diversity (dissimilarity between samples) have been observed in manipulative experiments [170, 174], as well as under natural perturbations [41]. This observation is consistent with the recently proposed “Anna Karenina principle,” which suggests that intraspecific variability is higher in dysbiotic than in healthy individuals [175]. In terms of function, dysbiosis has been correlated with an enrichment of cell motility, chemotaxis, or virulence genes in stressed tissues compared to healthy controls [158, 176].

Stress may also compromise host physiology and immunity [177–179], entailing loss of control over the microbiome; thus, dysbiosis could be responsive rather than causal. To date, few studies have investigated the molecular response of the sponge host upon perturbation, and they have mainly focused on thermal stress in the sponge host using real-time quantitative PCR [158, 171, 172, 180] or transcriptomics [181]. These studies showed that the *hsp70* gene as well as apoptosis-related, signaling, and oxidative stress-related genes are involved in sponge response to thermal stress. For example, host gene expression changes in *Rhopaloeides odorabile* were observed at sublethal temperatures (31 °C) [172], as well as at lethal temperatures (32 °C) that coincided with necrosis [158]. The downregulation of oxidative stress-related and signaling genes, such as glutathione-S-transferase and calmodulin, in *R. odorabile* adults suggests fatal loss of function related to stress and was accompanied by dysbiosis [158, 182]. Additionally, physiological stress in sponges has been assessed in response to ocean warming and sedimentation by monitoring respiration, nutrient fluxes, or lipid content [168, 170, 183]. Responses were highly variable, species-specific, and dependent on the duration and strength of the treatment. Unlike in cnidarians [184–186], sponge immune ecology (i.e., patterns of immune gene expression along natural gradients and under environmental stress) remains largely unexplored and the link between differential gene expression levels and physiology is still missing.

### Diseases

We are witnessing an unprecedented increase of disease and disease-like syndromes affecting a range of benthic organisms, including sponges, corals, and algae [187–189], some of which are resulting in recurrent mass mortality events [190]. The underlying causes are mostly unknown, but disease outbreaks seem to respond to multiple factors, such as cumulative environmental pressures that trigger physiological stress and the proliferation of opportunistic, as well as pathogenic microbes [191–193]. As for humans [163], dysbiosis has been proposed as an additional explanation for the increased susceptibility of marine organisms to disease [164].

In sponges, disease outbreaks resulting in drastically decimated population sizes have been reported worldwide [189]. In the Mediterranean Sea, 80–95% of *Ircinia fasciculata* and *Sarcotragus spinosulum* specimens died in the summers of 2008 and 2009 [194, 195]. In the Great Barrier Reef, a widespread distribution of a disease-like syndrome characterized by brown spot lesions and tissue necrosis has been observed in *Ianthella basta*, a common Indo-Pacific sponge species [196, 197]. Isolating and identifying causative agents has been unsuccessful so far [198, 199] (with the exception of pathogenic *Pseudoalteromonas agarivorans* strain NW4327 found in diseased Great Barrier Reef sponge *Rhopaloeides odorabile* [200, 201]). However, in many of these studies, diseased specimens showed divergent microbial profiles compared with the healthy individuals [202–206]. For example, diseased individuals of the deep-sea sponge *Geodia barretti* showed higher relative abundances of *Bacteroidetes*, *Firmicutes*, and *Deltaproteobacteria* than healthy ones [204], whereas in the Mediterranean sponge *Ircinia fasciculata* [202], the early-diseased (i.e., near to necrotic) tissue showed enrichment of *Gammaproteobacteria* and *Acidobacteria* groups but depletion of *Deltaproteobacteria*. However, in both cases, the sponge-associated microbiota shifted from a specific- to a generalist-dominated community in the unhealthy individuals. These findings indicate that, similar to corals [207, 208], sponge diseases appear to start with an imbalance of the holobiont which is then followed by opportunistic or polymicrobial infections.

### Acclimatization and adaptation: when change is good

Alterations in the symbiotic microbial community upon environmental stress can potentially lead to holobiont acclimatization and even adaptation [165]. Although the host can also respond to perturbations through phenotypic plasticity [209], microbial-mediated acclimatization has received special attention since microorganisms have shorter generation times and accordingly respond much more rapidly and versatilely than the host itself [210]. The microbial genetic information can either change through the introduction of new microorganisms from the environment or by genetic alteration of the associated microbiome through mutation and/or HGT leading to the acquisition of novel functions without shifts in taxonomic composition [211, 212]. Hence, novel acquired traits and functions in the microbiome could significantly affect the holobiont phenotype leading to acclimatization. If those new traits are vertically transmitted, they will facilitate microbiome-mediated transgenerational acclimatization upon which selection could act potentially leading to holobiont adaptation [165].

A recent study suggests that changes in the microbial community contribute to the ability of the sponge holobiont to cope with environmental change [213]. The effect of ocean acidification was assessed in three ubiquitous Mediterranean sponges (*Dysidea avara*, *Agelas oroides*, and *Chondrosia reniformis*) [213]. While the overall microbial abundance, richness, and diversity were not affected, species-specific differences in the acquisition of new microbes were observed: high acquisition in *D. avara*, moderate in *A. oroides*, and null in *C. reniformis*. This variation in microbial acquisition was inversely correlated with growth rate as growth was not affected in *D. avara,* reduced in *A. oroides* and severely reduced in *C. reniformis*. These results, together with evidence from coral holobionts [210, 214], suggest that symbiotic microbes influence the holobiont’s capacity to acclimate to changing environmental conditions.

### Sponges under future-ocean scenarios

It has been hypothesized that sponges may be “winners” under projected global change scenarios compared with other benthic invertebrates like corals [215]. Increased sponge abundances have been observed in some habitats [215], but they are often linked to proliferation of only a few or single species and thus accompanied by an overall loss of species diversity [216–218]. Other studies have documented localized losses in both sponge diversity and abundances [219] and for many more habitats, particularly in deep-sea sponge grounds, we lack baseline data [26, 218]. Sponge mass mortality events in response to environmental perturbations [191, 220] combined with the results from experimental studies (Table 2) indicate that sponge diversity and function will change in the future, with unknown cascading ecosystem effects. Moreover, baseline data and diagnostic tools to detect these changes are lacking [221]. Microbial monitoring was proposed as a diagnostic tool since microbes potentially serve as early warning indicators for stress at both the holobiont and the ecosystem level [164, 222]. In combination with traditional ecological monitoring programs, microbial monitoring would allow us (i) to acquire missing baseline data, (ii) to predict approaching tipping points, and (iii) to identify long-term trends that may inform management [223, 224]. Ultimately, this would enable intervention before the key ecosystem functions provided by sponges are lost.

## Conclusion

In this review, we have examined the sponge holobiont from the micro- to the global scale. Advances in sponge microbiology have revealed the principles of diversity and core functions, but linking microbial diversity with function would provide additional insights into sponge holobiont health. Sponges illustrate the concept of nested ecosystems, providing a new framework for understanding holobionts in the marine environment. Future research should attempt to connect these multiple scales in order to understand which microbial features contribute to sponge holobiont functioning and to what extent they impact the surrounding ecosystem. The response of the holobiont to environmental stress requires the evaluation of both host and microbiota in a true holobiont approach. Defining the relationship between stress, dysbiosis, and disease requires moving beyond patterns to mechanisms that can establish cause and consequence. Only then can we disentangle the underlying principles of health in sponge holobionts, improve predictions of the fate of sponges in future ocean-scenarios, and develop effective management strategies accordingly.

## Box 1 Glossary

**Acclimatization:** The capacity of a holobiont to adjust to a perturbation through host phenotypic plasticity or restructuring of the microbiome in order to reach a new stable state

**Adaptation:** A transgenerational process that enhances the fitness of the holobiont through transgenerational acclimatization, heritable microbial community changes, or host/symbiont evolution

**Core microbiome:** The set of microbial taxa which are consistently and stably prevalent in host individuals of the same species

**Dysbiosis:** The divergence of a symbiotic microbial community from the community found in healthy individuals

**Disease:** The impairment of normal function following perturbation or damage. May be, but is not necessarily, induced by a pathogenic microorganism

**Functional convergence:** In the holobiont context, symbiotic microbial communities with different evolutionary histories that have, via different but analogous pathways, converged upon similar functional solutions

**Functional redundancy:** The presence of several microbial taxa within an ecosystem or holobiont that perform the same functions, such that the loss of one particular taxon or a shift in the community diversity would not compromise ecosystem function

**Holobiont health:** A dynamic equilibrium that allows minor fluctuations in terms of diversity or functions to ensure the maintenance of symbiotic homeostasis

**Microbiota:** The assemblage of microorganisms present in a defined environment or host

**Microbiome:** The group of microbes, their genetic information, and the surrounding environmental conditions in a defined environment or host

**Nested ecosystem**: A smaller distinct ecosystem which is contained within and interacts with a larger ecosystem or series of successively larger ecosystems

**Opportunistic:** An organism that is capable of causing damage to a host under specific conditions, but may also exist as a commensal within the same host under normal conditions

**Perturbation:** A temporary or persistent change in biotic or abiotic conditions that leads to a response by an ecosystem or holobiont

**Resilience:** The capacity of a system to recover its initial functional and taxonomical composition and return to an initial stable state following a perturbation

**Resistance:** The property of a system to remain unchanged and maintain at a stable state upon perturbation

**Symbiosis (sensu De Bary):** The close association of two or more organisms of a different species. This association may be mutualistic, commensal, or parasitic

## Box 2 The HMA-LMA dichotomy

Sponges can be classified into two groups according to the abundance and density of microbes in their tissues. High microbial abundance (HMA) sponges harbor densities of microbes 2–4 orders of magnitude higher than low microbial abundance (LMA) sponges [225, 226]. A recent publication made use of the Global Sponge Microbiome Project data to further investigate the microbial diversity features of HMA and LMA sponges at large scale by way of a machine learning [227]. HMA sponges harbor richer and more diverse microbial communities than LMA sponges [227] (although there are few exceptions to this pattern, e.g., [228]). Additionally, certain taxa (from phylum to 97% OTU-level resolution) are significantly enriched in either one or the other group [227]. For example, LMA sponges are enriched in *Proteobacteria* and *Cyanobacteria* whereas HMA sponges are enriched in *Chloroflexi*, *Acidobacteria*, or *Poribacteria*, among others. Despite these differences in microbial diversity and abundance, the functional convergence of microbiome core functions appears to span the HMA-LMA dichotomy [31, 53]. However, differences in gene abundances between central metabolic functions of LMA and HMA microbial communities have been reported [64]. Interestingly, sponge species diverge in presenting one of these two microbial configurations, regardless of host phylogeny [226, 227]. It has been proposed that sponge morphology may be a determinant factor: HMA sponges have denser tissues with less-developed water channels compared with LMA sponges [226, 229, 230]. Still, the processes underlying this dichotomy remain unknown.

## Box 3 Future directions in sponge holobiont research

**1. Adopt a true holobiont approach**. Define and elucidate the functional roles of the missing holobiont members. Microbial groups other than bacterial and archaea, such as viruses or microbial eukaryotes, remain understudied. Advance the understanding of the drivers of microbial assembly and microbe-microbe interactions. Define the role of the host as an ecosystem engineer and determine the mechanisms underpinning host-microbe interactions.

**2. Integrate the concept of nested ecosystems into holobiont research**. Validate and quantify the influence of the sponge microbiome at the holobiont, community, and ecosystem scale. Determine how environmental factors can alter microbiome-mediated processes and link mechanisms at multiple scales. It will also be important to consider that different sponge holobionts fulfill different functions and that functioning varies across environments. Research has largely focused on shallow-water ecosystems while the deep-sea remains understudied.

**3. Determine the drivers and processes governing holobiont health and stability**. Unravel the relationships between diversity, function, and holobiont stability and establish the mechanisms behind resistance and resilience. Determine the role of functional redundancy in contributing to holobiont stability. Disentangle the linkages between stress, dysbiosis, and disease in sponge holobionts and elucidate whether dysbiosis is a cause or consequence of disease. Evaluate the role of microbes in mediating holobiont acclimatization and adaptation to environmental change.

**4. Monitoring, management, and solutions**. Develop long-term monitoring strategies to collect missing baseline data and identify long-term trends. Assess the vulnerability of sponge holobionts to global change and other anthropogenic stressors, and develop management solutions to ensure the maintenance of sponge holobiont functions at the ecosystem level. This will entail multidisciplinary approaches that combine experimental, field, and genomic/transcriptomic data.

## Abbreviations

CRISPR: Clustered regularly interspaced short palindromic repeats
DGGE: Denaturing gradient gel electrophoresis
DIN: Dissolved inorganic nitrogen
DOM: Dissolved organic matter
ELP: Eukaryotic-like proteins
EMP: Earth microbiome project
HGT: Horizontal gene transfer
LPS: Lipopolysaccharide
POM: Particulate organic matter
PRR: Pattern recognition receptor
RFLP: Restriction fragment length polymorphism
RT-qPCR: Real-time quantitative polymerase chain reaction
SRCR: Scavenger receptor cysteine-rich
TRFLP: Terminal restriction fragment length polymorphism
